# Supplementary material for: Non-invasive assessment of telomere maintenance mechanisms in brain tumors
Source: Nat Commun. 2021 Jan 4;12:92. doi: 10.1038/s41467-020-20312-y (PMC7782549; doi:10.1038/s41467-020-20312-y)
Supplement: Supplementary file 1 — Supplementary Information [file 41467_2020_20312_MOESM1_ESM.pdf]

Supplementary Table 1

	IDHmut and TMM status					
	**IDHmut expression**	**TERT expression**	**Telomerase activity**	**ATRX expression**	**c-circles**	**TSCE**
NHA$_{CONTROL}$	Yes[13,35]	No	No	Yes	No	No[13]
NHA$_{TERT}$	Yes[35]	Yes	Yes	Yes	No	ND
NHA$_{ALT}$	Yes[13]	No	No	No	Yes	Yes[13]
NHA$_{TERT+ IDHmut-}$	No[32,35]	Yes	Yes	Yes	No	ND
NHA$_{ATRX- IDHmut-}$	No[13]	No	No	No	No	No[13]
BT54	Yes[44-46]	Yes	Yes	Yes	No	ND
SF10417	Yes[6,54]	Yes	Yes	Yes	No	ND
BT142 ALT+	Yes[47]	No	No	No	Yes	Yes (Supplementary Fig. 1g)
BT142 ALT-	Yes[47]	No	No	Yes	No	No (Supplementary Fig. 1g)
BT142 TERT+	Yes[47]	Yes	Yes	No	No	ND
MGG119 ALT+	Yes[51]	No	No	No	Yes	Yes (Supplementary Fig. 1f)
MGG119 TERT+	Yes[51]	Yes	Yes	No	No	No (Supplementary Fig. 1f)

Supplementary Table 1. Summary of cell lines used in this study. The IDHmut status of all the models in this study has previously been examined (superscripts indicate the relevant references). For TERT expression data as measured by RT-PCR, see Supplementary Fig. 1a. Results of telomerase activity are presented in Supplementary Fig. 1b. ATRX expression data is presented in Supplementary Fig. 1c. Quantification of c-circle levels is presented in Supplementary Fig. 1e. Verification of T-SCE has been previously described (refer to the superscripts for the relevant references) or is presented in Supplementary Fig. 1f-1g. ND = not determined.

Supplementary Table 2

	Primer sequences used in this study	
**Gene**	**Forward primer**	**Reverse Primer**
TERT	TCACGGAGACCACGTTTCAAA	TTCAAGTGCTGTCTGATTCCAAT
ATRX	TCCTTGCACACTCATCAGAAGAATC	CGTGACGATCCTGAAGACTTGG
GLS1	TGGACTATGAAAGTCTCCAACAAGA	CTCATTTGACTCAGGTGACACTTTT
NAMPT	GTTCCAGCAGCAGAACACAG	GCTGACCACAGATACAGGCA
ASCT2	CGGCACGCCCGGGAGGCTTTC	GAATCTGGGGGCCGGGAAGCGG
LAT2	TTGCCAATGTCGCTTATGTCA	AGAGACCCATTAACTCCTCCAAA
ACTB	AGAGCTACGAGCTGCCTGAC	AGCACTGTGTTGGCGTACAG

Supplementary Table 2. List of primer sequences used in this study.

Supplementary figure 1

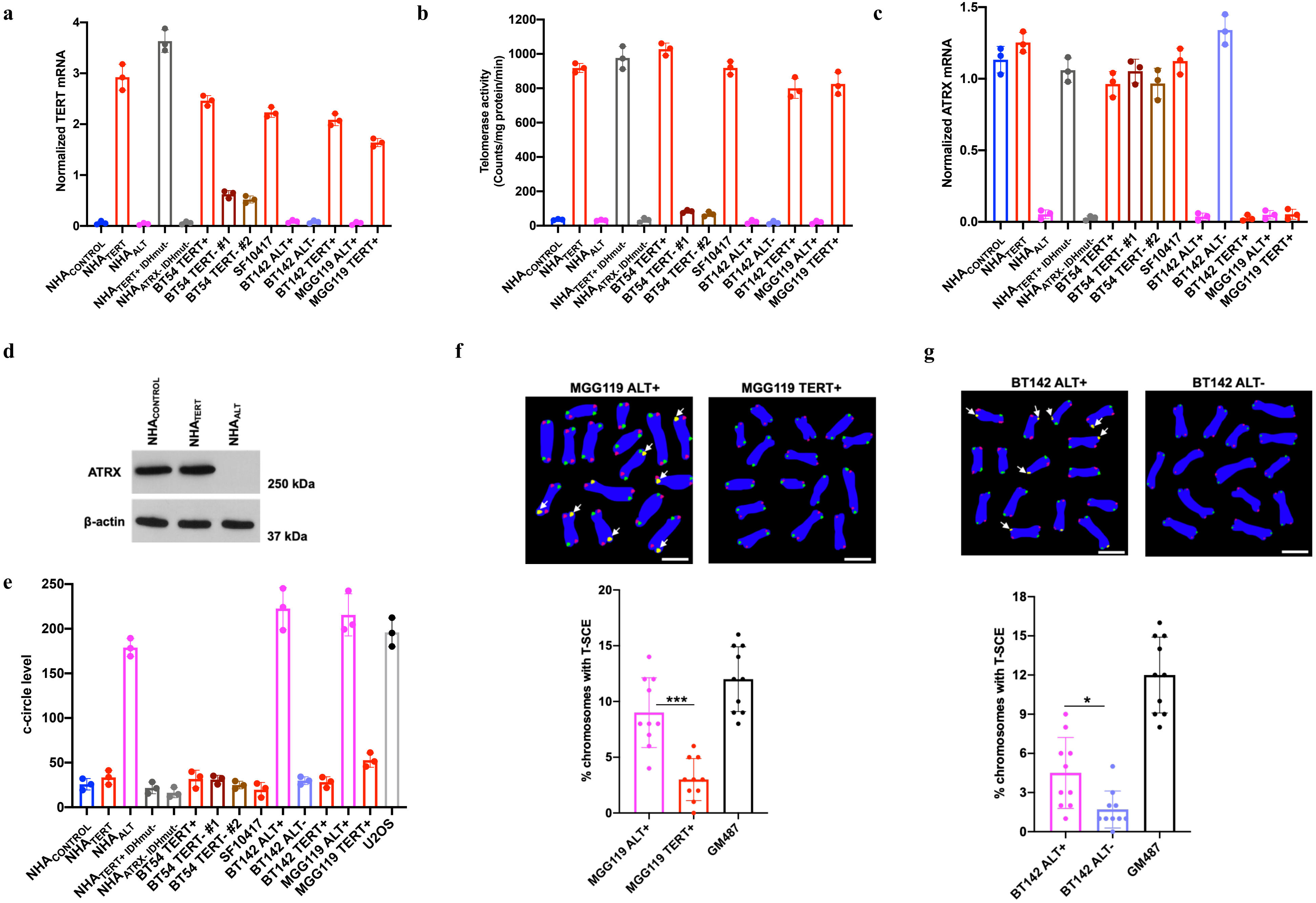

Supplementary Figure 1. Characterization of the TMM status of the cell lines used in this study. TERT expression as measured by quantitative RT-PCR **(a)**, telomerase activity as measured by the TRAP assay **(b)** and ATRX expression as measured by quantitative RT-PCR **(c)** in the genetically engineered and patient-derived LGOG and LGA models used in this study (n=3 each). (**d**) Assessment of ATRX expression in $NHA_{CONTROL}$, NHA_{TERT} and NHA_{ALT} models by western blotting (n=3 each, please refer to source data file for uncropped blots). **(e)** Quantification of c-circle levels in the models in this study (n=3 each). **(f)** Top panel: T-SCE as assessed by colocalization of leading (red)- and lagging (green)-strand telomeric probes in MGG119 ALT+ (magenta circles) and MGG119 TERT+ (red circles) neurospheres. The presence of yellow foci (highlighted with arrows) points to the occurrence of T-SCE. Bottom panel: quantification of the percentage of chromosomes with T-SCE (n=10 each). Scale bar represents 1 µm. (**g**) Top panel: T-SCE as assessed by colocalization of leading (red)- and lagging (green)-strand telomeric probes in BT142 ALT+ (magenta circles) and BT142 ALT- (lavender circles) neurospheres. The presence of yellow foci (highlighted with arrows) points to the occurrence of T-SCE. Bottom panel: quantification of the percentage of chromosomes with T-SCE (n=10 each). Scale bar represents 1 µm. Data are presented as mean ± standard deviation. Statistical significance was assessed using an unpaired Student's t-test assuming unequal variance with $p<0.05$ considered significant. Source data and exact p values, where applicable, are provided as a source data file.

Supplementary figure 2

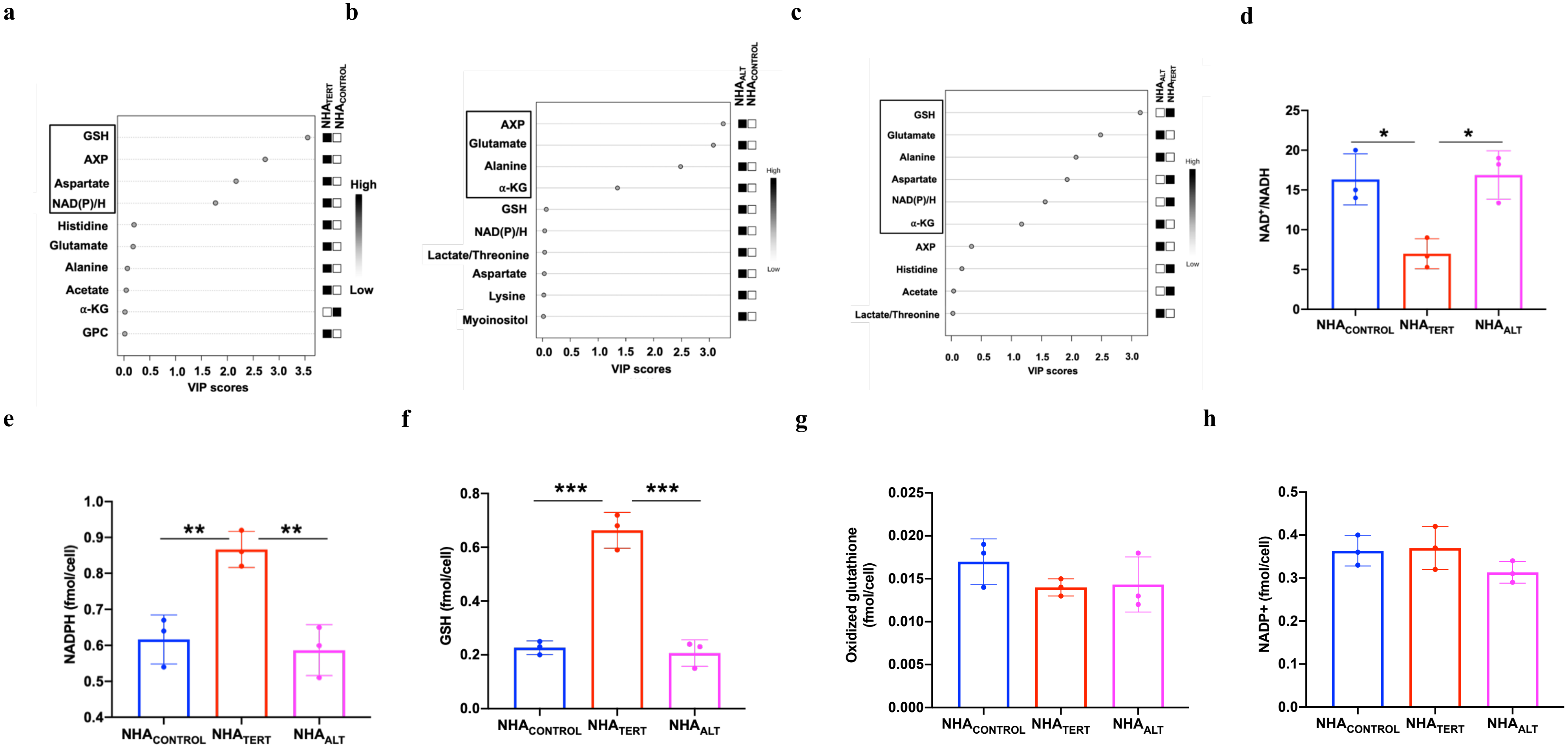

Supplementary Figure 2. TERT expression and the ALT pathway are linked to unique patterns of metabolic reprogramming in low-grade glioma cells. (a) VIP scores from PLSDA analysis for $NHA_{CONTROL}$ and NHA_{TERT} cells (n = 5 biological replicates each). GPC: glycerophosphocholine. **(b)** VIP scores from PLSDA analysis for $NHA_{CONTROL}$ and NHA_{ALT} cells (n = 5 biological replicates each). **(c)** VIP scores from PLSDA analysis for NHA_{TERT} and NHA_{ALT} cells (n = 5 biological replicates each). NAD^+/NADH ratio (**d**), NADPH **(e)**, GSH **(f)**, oxidized glutathione **(g)** and $NADP^+$ **(h)** as measured by spectrophotometric assays in $NHA_{CONTROL}$ (blue circles), NHA_{TERT} (red circles) and NHA_{ALT} (magenta circles) models (n = 3 biological replicates each). Data are presented as mean ± standard deviation. Statistical significance was assessed using an unpaired Student's t-test assuming unequal variance with $p<0.05$ considered significant. *** represents statistical significance with $p < 0.005$; ** represents statistical significance with $p < 0.01$; * represents statistical significance with $p < 0.05$. Source data with exact p values are provided as a source data file.

Supplementary figure 3

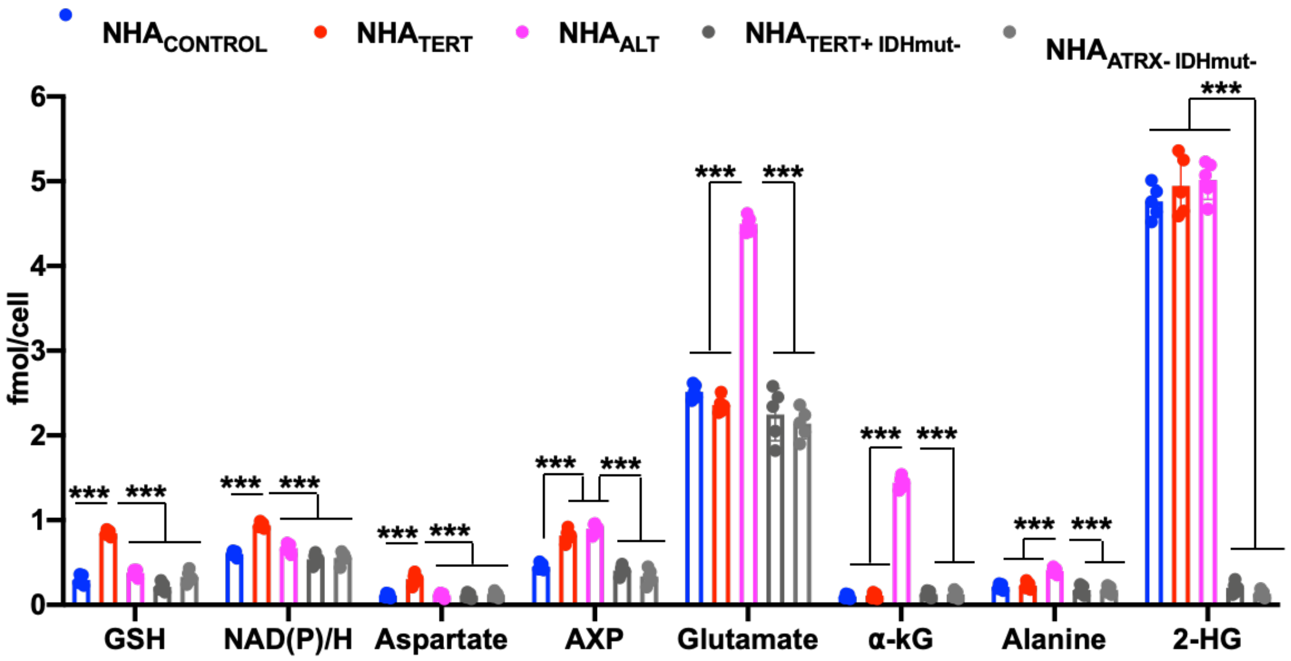

Supplementary Figure 3. Assessment of the contributions of IDHmut, ATRX and TERT expression to TMM-linked metabolic biomarkers. Steady-state metabolite levels as measured by ^{1}H-MRS in $NHA_{CONTROL}$ (blue circles), NHA_{TERT} (red circles), NHA_{ALT} (magenta circles), $NHA_{TERT+IDHmut-}$ (dark grey circles) and $NHA_{ATRX-IDHmut-}$ (light grey circles) models (n = 5 biological replicates for each model). Data are presented as mean ± standard deviation. Statistical significance was assessed using an unpaired Student's t-test assuming unequal variance with $p<0.05$ considered significant. Correction for multiple comparisons was performed using the Holm-Šídák method. *** represents statistical significance with $p < 0.005$. Source data with exact p values are provided as a source data file.

Supplementary figure 4

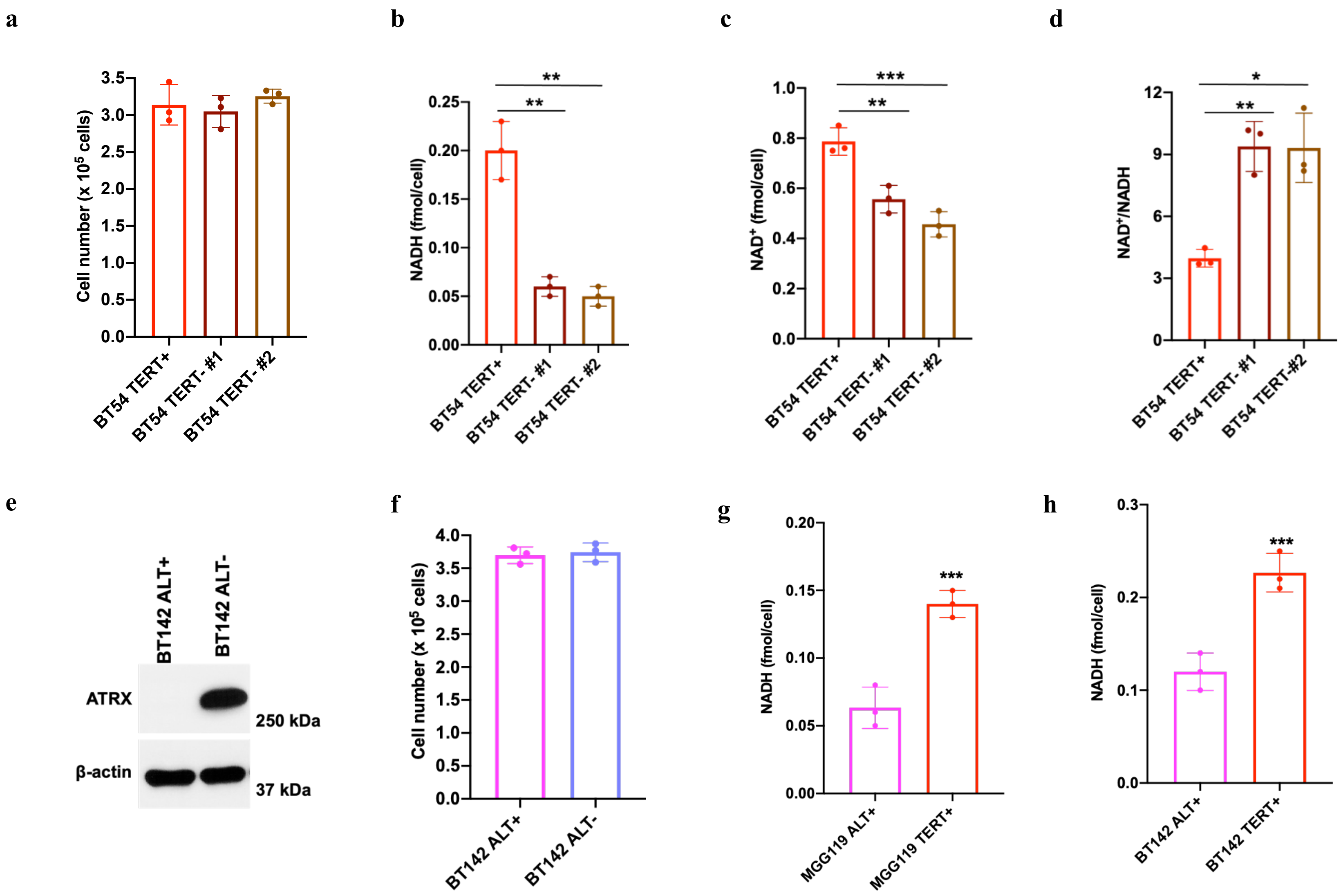

Supplementary Figure 4. TERT expression and the ALT pathway are linked to MRS-detectable metabolic alterations in patient-derived LGOG and LGA models. (a) Effect of silencing *TERT* on cell proliferation in the BT54 LGOG model. *TERT* was silenced by RNA interference using 2 independent siRNA sequences/pools. NADH **(b)**, NAD^+ **(c)** and the NAD^+/NADH ratio **(d)** in BT54 TERT+ (red circles), BT54 TERT- #1 (dark brown circles) and BT54 TERT- #2 (light brow circles) neurospheres. **(e)** ATRX protein levels as determined by immunoblotting in BT142 ALT+ and BT142 ALT- neurospheres. **(f)** Effect of silencing the ALT pathway via *ATRX* re-expression on cell proliferation in the BT142 model. NADH levels as measured by spectrophotometry in isogenic ALT+ (magenta circles) and TERT+ (red circles) neurospheres in the MGG119 **(g)** and BT142 **(h)** models. All experiments were performed on 3 biological replicates (n = 3). Data are presented as mean ± standard deviation. Statistical significance was assessed using an unpaired Student's t-test assuming unequal variance with $p<0.05$ considered significant. *** represents statistical significance with $p < 0.005$; ** represents statistical significance with $p < 0.01$; * represents statistical significance with $p < 0.05$. Source data with exact p values are provided as a source data file.

Supplementary figure 5

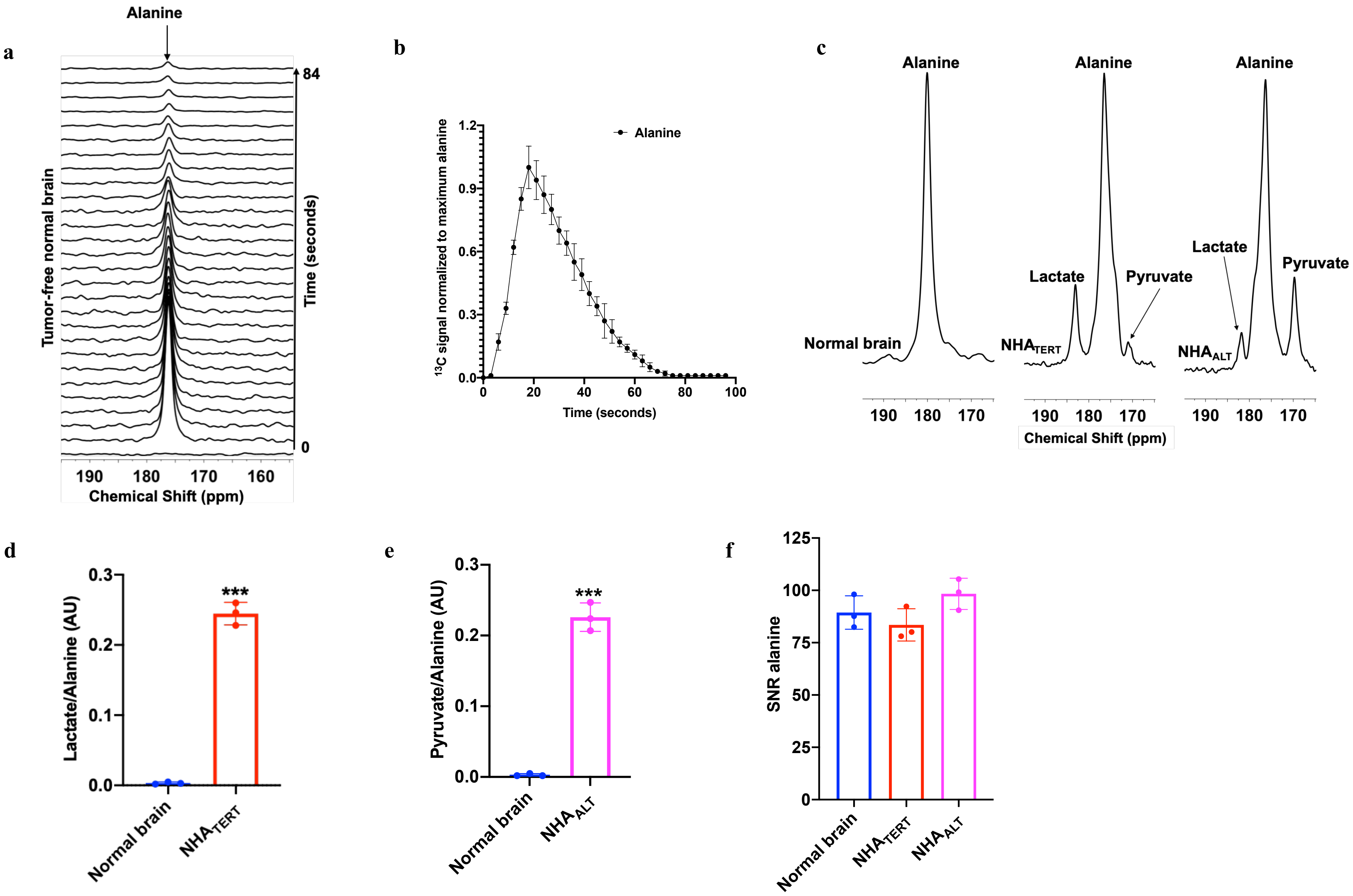

Supplementary Figure 5. Hyperpolarized [1-^{13}C]-alanine can non-invasively monitor TMM status *in vivo*. (a) Representative array of dynamic ^{13}C-MRS spectra acquired from a 15 mm slab following intravenous injection of hyperpolarized [1-^{13}C]-alanine into a tumor-free, healthy control rat. **(b)** Quantification of the build-up of hyperpolarized [1-^{13}C]-alanine in healthy, tumor-free control rats. **(c)** Representative summed ^{13}C-MRS spectra showing hyperpolarized [1-^{13}C]-alanine metabolism in tumor-free healthy control rats and rats bearing orthotopic NHA_{TERT} and NHA_{ALT} tumor xenografts. **(d)** Comparison of the hyperpolarized lactate/alanine ratio in tumor-free healthy control rats (blue circles) vs. rats bearing orthotopic NHA_{TERT} (red circles) tumors. **(e)** Quantification of the hyperpolarized pyruvate/alanine ratio in tumor-free healthy control rats (blue circles) vs. rats bearing orthotopic NHA_{ALT} (magenta circles) tumors. **(f)** Quantification of the SNR of hyperpolarized [1-^{13}C]-alanine in tumor-free healthy control rats (blue circles) and rats bearing orthotopic NHA_{TERT} (red circles) and NHA_{ALT} (magenta circles) tumor xenografts. All experiments were performed on 3 biological replicates (n = 3). Data are presented as mean ± standard deviation. Statistical significance was assessed using an unpaired Student's t-test assuming unequal variance with $p<0.05$ considered significant. *** represents statistical significance with $p < 0.005$. Source data with exact p values are provided as a source data file.

Supplementary figure 6

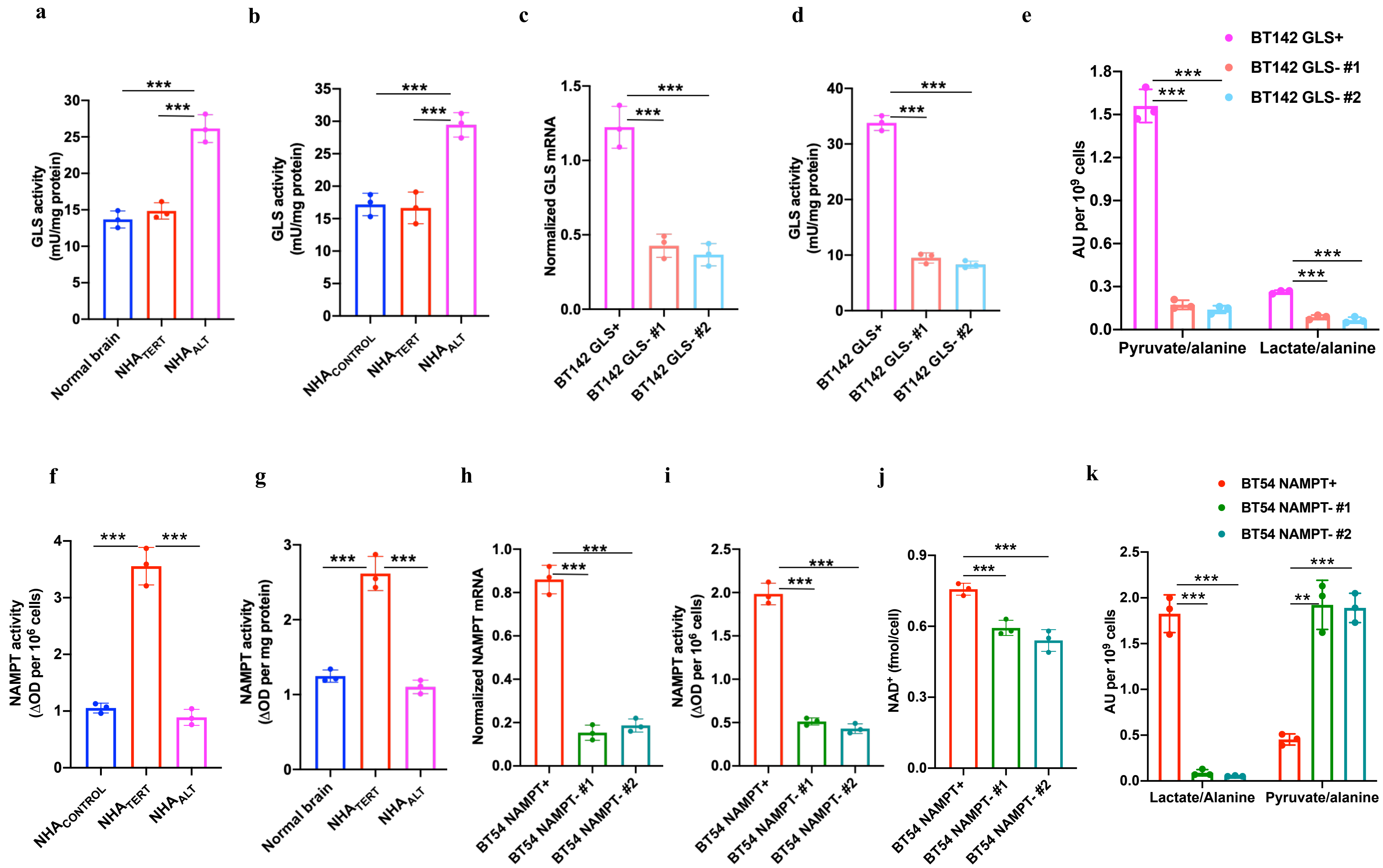

Supplementary Figure 6. GLS1, NAMPT, ASCT2 and LAT2 mechanistically link TERT expression and the ALT pathway to metabolic alterations in LGOG and LGA models. (a) GLS activity in $NHA_{CONTROL}$ (blue circles), NHA_{TERT} (red circles) and NHA_{ALT} (magenta circles) cells. **(b)** GLS activity in *ex vivo* tissue samples from tumor-free healthy controls rats (blue circles) and rats bearing orthotopic NHA_{TERT} (red circles) and NHA_{ALT} (magenta circles) tumor xenografts. Verification of GLS1 knock-down by quantitative RT-PCR for *GLS1* mRNA **(c)** and GLS activity **(d)** in BT142 GLS+ (magenta circles), BT142 GLS- #1 (orange circles) and BT142 GLS- #2 (cyan circles) neurospheres. *GLS1* silencing was performed using two independent siRNA sequences/pools. **(e)** Hyperpolarized pyruvate/alanine and lactate/alanine ratios in BT142 GLS+ (magenta circles), BT142 GLS- #1 (orange circles) and BT142 GLS- #2 (cyan circles) neurospheres. **(f)** NAMPT activity in $NHA_{CONTROL}$, NHA_{TERT} and NHA_{ALT} cells. **(g)** NAMPT activity in *ex vivo* tissue samples from tumor-free healthy controls rats (blue circles) and rats bearing orthotopic NHA_{TERT} (red circles) and NHA_{ALT} (magenta circles) tumor xenografts. Verification of NAMPT knock-down by quantitative RT-PCR for *NAMPT* mRNA **(h)** and NAMPT activity **(i)** in BT54 NAMPT+ (red circles), BT54 NAMPT- #1 (green circles) and BT54 NAMPT- #2 (teal circles) neurospheres. *NAMPT* silencing was achieved by using two independent siRNA sequences/pools. **(j)** NAD^+ levels in BT54 NAMPT+ (red circles), BT54 NAMPT- #1 (green circles) and BT54 NAMPT- #2 (teal circles) neurospheres. **(k)** Ratios of hyperpolarized lactate/alanine and pyruvate/alanine in BT54 NAMPT+ (red circles), BT54 NAMPT- #1 (green circles) and BT54 NAMPT- #2 (teal circles) neurospheres. All experiments were performed on 3 biological replicates (n = 3). Data are presented as mean ± standard deviation. Statistical significance was assessed using an unpaired Student's t-test assuming unequal variance with $p<0.05$ considered significant. *** represents statistical significance with $p < 0.005$; ** represents statistical significance with $p < 0.01$. Source data with exact p values are provided as a source data file.

Supplementary figure 7

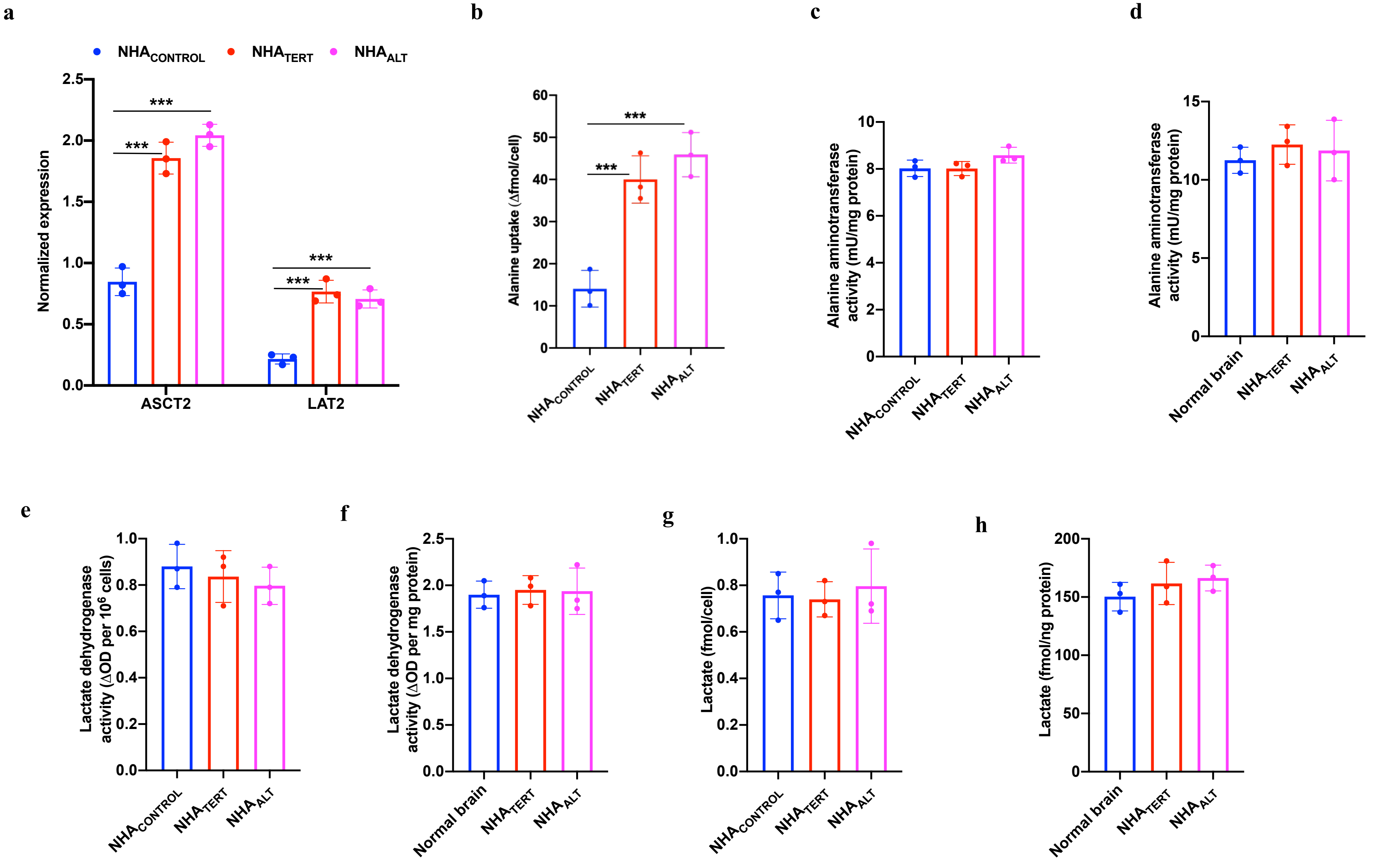

Supplementary Figure 7. Analysis of factors involved in hyperpolarized [1-^{13}C]-alanine metabolism in LGOG and LGA models. (a) Expression of *ASCT2* and *LAT2* as determined by quantitative RT-PCR in $NHA_{CONTROL}$ (blue circles), NHA_{TERT} (red circles) and NHA_{ALT} (magenta circles) models. **(b)** [1-^{13}C]-alanine uptake in $NHA_{CONTROL}$ (blue circles), NHA_{TERT} (red circles) and NHA_{ALT} (magenta circles) cells. **(c)** Alanine aminotransferase activity in $NHA_{CONTROL}$ (blue circles), NHA_{TERT} (red circles) and NHA_{ALT} (magenta circles) cells. **(d)** Alanine aminotransferase activity in normal brain (blue circles), NHA_{TERT} (red circles) and NHA_{ALT} (magenta circles) tumor xenografts. **(e)** Lactate dehydrogenase activity in $NHA_{CONTROL}$ (blue circles), NHA_{TERT} (red circles) and NHA_{ALT} (magenta circles) cells. **(f)** Lactate dehydrogenase activity in normal brain (blue circles), NHA_{TERT} (red circles) and NHA_{ALT} (magenta circles) tumor xenografts. **(g)** Steady-state lactate levels measured by ^{1}H-MRS in $NHA_{CONTROL}$ (blue circles), NHA_{TERT} (red circles) and NHA_{ALT} (magenta circles) cells. **(h)** Steady-state lactate measured by ^{1}H-MRS in normal brain (blue circles), NHA_{TERT} (red circles) and NHA_{ALT} (magenta circles) tumor xenografts. All experiments were performed on 3 biological replicates ($n = 3$). Data are presented as mean ± standard deviation. Statistical significance was assessed using an unpaired Student's t-test assuming unequal variance with $p<0.05$ considered significant. Correction for multiple comparisons was performed using the Holm-Šídák method. *** represents statistical significance with $p < 0.005$. Source data are provided as a source data file.
