# Supplementary material for: Non-invasive assessment of telomere maintenance mechanisms in brain tumors
Source: Nat Commun. 2021 Jan 4;12:92. doi: 10.1038/s41467-020-20312-y (PMC7782549; doi:10.1038/s41467-020-20312-y)
Supplement: Supplementary file 3 — Description of Additional Supplementary Files [file 41467_2020_20312_MOESM3_ESM.pdf]

Description of Additional Supplementary Files

Supplementary Software: The Supplementary software available with this manuscript and at the Viswanath laboratory Github repository (https://github.com/ViswanathLab/EPSI) includes custom Matlab (Matlab version 2018a) codes we have used for analysis of 2D echo planar spectroscopic imaging (EPSI) data from our hyperpolarized ^{13}C-MRS studies in preclinical brain tumor models.
