# Supplementary material for: Non-invasive assessment of telomere maintenance mechanisms in brain tumors
Source: Nat Commun. 2021 Jan 4;12:92. doi: 10.1038/s41467-020-20312-y (PMC7782549; doi:10.1038/s41467-020-20312-y)
Supplement: Supplementary file 5 — Reporting Summary [file 41467_2020_20312_MOESM5_ESM.pdf]

nature research

Corresponding author(s): PAVITHRA VISWANATH, SABRINA RONEN

Last updated by author(s): Nov 16, 2020

Reporting Summary

Nature Research wishes to improve the reproducibility of the work that we publish. This form provides structure for consistency and transparency in reporting. For further information on Nature Research policies, see our Editorial Policies and the Editorial Policy Checklist.

Statistics

For all statistical analyses, confirm that the following items are present in the figure legend, table legend, main text, or Methods section.

n/a	Confirmed	
☐	☒	The exact sample size (*n*) for each experimental group/condition, given as a discrete number and unit of measurement
☐	☒	A statement on whether measurements were taken from distinct samples or whether the same sample was measured repeatedly
☐	☒	The statistical test(s) used AND whether they are one- or two-sided<br>*Only common tests should be described solely by name; describe more complex techniques in the Methods section.*
☒	☐	A description of all covariates tested
☐	☒	A description of any assumptions or corrections, such as tests of normality and adjustment for multiple comparisons
☐	☒	A full description of the statistical parameters including central tendency (e.g. means) or other basic estimates (e.g. regression coefficient) AND variation (e.g. standard deviation) or associated estimates of uncertainty (e.g. confidence intervals)
☐	☒	For null hypothesis testing, the test statistic (e.g. *F*, *t*, *r*) with confidence intervals, effect sizes, degrees of freedom and *P* value noted<br>*Give P values as exact values whenever suitable.*
☒	☐	For Bayesian analysis, information on the choice of priors and Markov chain Monte Carlo settings
☒	☐	For hierarchical and complex designs, identification of the appropriate level for tests and full reporting of outcomes
☒	☐	Estimates of effect sizes (e.g. Cohen's *d*, Pearson's *r*), indicating how they were calculated

Our web collection on statistics for biologists contains articles on many of the points above.

Software and code

Policy information about availability of computer code

Data collection	Bruker Biospin 3T Paravision version 6.
Data analysis	Mestrenova version 14.1.2, Metaboanalyst R3.0, Microsoft Excel version 16, GraphPad Prism 9. The imaging data in this manuscript was analyzed using custom Matlab codes (Matlab version 2018a). The codes with operating instructions are available with this manuscript and at a GitHub repository (https://github.com/ViswanathLab/EPSI).

For manuscripts utilizing custom algorithms or software that are central to the research but not yet described in published literature, software must be made available to editors and reviewers. We strongly encourage code deposition in a community repository (e.g. GitHub). See the Nature Research guidelines for submitting code & software for further information.

Data

Policy information about availability of data

All manuscripts must include a data availability statement. This statement should provide the following information, where applicable:

- Accession codes, unique identifiers, or web links for publicly available datasets
- A list of figures that have associated raw data
- A description of any restrictions on data availability

There are no accession codes, unique identifiers or publicly available datasets associated with this manuscript. Source data underlying all the findings in this study are available as a source data file. All other data that support the findings of this study are available within the manuscript, its Supplementary Information files, and from the corresponding author (PV) upon reasonable request. A detailed data availability statement has been provided in the manuscript.

Field-specific reporting

Please select the one below that is the best fit for your research. If you are not sure, read the appropriate sections before making your selection.

☒ Life sciences ☐ Behavioural & social sciences ☐ Ecological, evolutionary & environmental sciences

For a reference copy of the document with all sections, see nature.com/documents/nr-reporting-summary-flat.pdf

Life sciences study design

All studies must disclose on these points even when the disclosure is negative.

Sample size	Sample sizes for all experiments were chosen empirically. Statistical significance was assessed using an unpaired Student's T-test assuming unequal variance with $p<0.05$ considered significant. Where applicable, correction for multiple comparisons was performed using the Holm-Šídák method.
Data exclusions	No data points were excluded in this study
Replication	All experiments were performed on a minimum of 3 biological replicates ($n\geq3$) tested on different occasions. In addition, in vivo experiments were independently performed by different personnel and all attempts at replication were confirmed to be successful.
Randomization	For all cell studies, batches of cells were randomly allocated into different test groups e.g. non-targeting siRNA control and gene-specific siRNA-treated groups. For animal studies, animals were randomized prior to tumor cell injection.
Blinding	Investigators were not blinded in this study. Our study involved experiments requiring very specialized expertise including the biological assays for verification of TERT or ALT status as well as the 1H- and hyperpolarized 13C-MRS experiments for identification of metabolic biomarkers. Therefore, identifying trained personnel who can perform these experiments in a blinded manner is difficult. Nevertheless, our studies have been independently verified by different personnel. In addition, since our in vivo studies compared tumor versus normal brain within individual animals, blinding was not necessary.

Reporting for specific materials, systems and methods

We require information from authors about some types of materials, experimental systems and methods used in many studies. Here, indicate whether each material, system or method listed is relevant to your study. If you are not sure if a list item applies to your research, read the appropriate section before selecting a response.

Materials & experimental systems

n/a	Involved in the study	
☐	☒	Antibodies
☐	☒	Eukaryotic cell lines
☒	☐	Palaeontology and archaeology
☐	☒	Animals and other organisms
☐	☒	Human research participants
☒	☐	Clinical data
☒	☐	Dual use research of concern

Methods

n/a	Involved in the study	
☒	☐	ChIP-seq
☒	☐	Flow cytometry
☒	☐	MRI-based neuroimaging

Antibodies

Antibodies used	ATRX antibody from Abcam (catalog # ab188027, clone number CL0537, 1:1000 dilution), β-actin (Cell Signaling, catalog # 4970, clone 13E5, 1:5000 dilution).
Validation	ATRX antibody was validated via knock-down studies performed by Abcam. ab188027 was shown to recognize ATRX in wild type cells as signal was lost at the expected MW in ATRX knockout cells. β-actin antibody was validated by Cell Signaling by via knock-out studies. Immunohistochemical analysis of paraffin-embedded human lung carcinoma using β-actin (13E5) Rabbit mAb in the presence of β-actin blocking Peptide #1025 showed loss of β-actin reactivity.

Eukaryotic cell lines

Policy information about cell lines

Cell line source(s)	Immortalized normal human astrocytes (NHAs) were originally obtained from Clonetics and engineered by the laboratory of Dr. Russell O Pieper. MGG119 cells are patient-derived tumor models that were engineered to be ALT+ or TERT+ by Dr. Pieper's group. BT142 and BT54 are patient-derived tumor models provided by the laboratory of Dr. Hema Artee Luchman. SF10417 cells are patient-derived LGOG cells that were provided by Dr. Joseph F Costello.

Authentication	All cell lines were routinely authenticated by short tandem repeat fingerprinting (Cell Line Genetics) and assayed within 6 months of authentication.
Mycoplasma contamination	All cell lines tested negative for mycoplasma contamination.
Commonly misidentified lines (See ICLAC register)	No commonly misidentified cell lines were used in this study.

Animals and other organisms

Policy information about studies involving animals; ARRIVE guidelines recommended for reporting animal research

Laboratory animals	This study used athymic nude rats from Envigo laboratories (male, rnu/rnu homozygous, 5-6-weeks old).
Wild animals	No wild animals were used in this study.
Field-collected samples	No field-collected samples were used in this study.
Ethics oversight	All animal studies were conducted in accordance with University of California Institutional Animal Care and Use Committee (IACUC) guidelines.

Note that full information on the approval of the study protocol must also be provided in the manuscript.

Human research participants

Policy information about studies involving human research participants

Population characteristics	No human subjects were involved in this study. We performed metabolic analysis on de-identified human biopsy tissues obtained in compliance with informed consent policy from the UCSF Brain Tumor Center Biorepository and Pathology Core. Sample use was approved by the Committee on Human Research at UCSF.
Recruitment	No human participants were involved in this study.
Ethics oversight	Committee on Human Research at UCSF

Note that full information on the approval of the study protocol must also be provided in the manuscript.
